# Health Effects of Electronic Cigarettes: An Umbrella Review and Methodological Considerations

**DOI:** 10.3390/ijerph19159054

**Published:** 2022-07-25

**Authors:** Nargiz Travis, Marie Knoll, Christopher J. Cadham, Steven Cook, Kenneth E. Warner, Nancy L. Fleischer, Clifford E. Douglas, Luz María Sánchez-Romero, Ritesh Mistry, Rafael Meza, Jana L. Hirschtick, David T. Levy

**Affiliations:** 1 Lombardi Comprehensive Cancer Center, Georgetown Medical University, Washington, DC 20057, USA; marieknoll18@gmail.com (M.K.); ls1364@georgetown.edu (L.M.S.-R.); dl777@georgetown.edu (D.T.L.); 2 Department of Health Management and Policy, School of Public Health, University of Michigan, Ann Arbor, MI 48109, USA; ccadham@umich.edu (C.J.C.); kwarner@umich.edu (K.E.W.); cdoug@umich.edu (C.E.D.); 3 Department of Epidemiology, School of Public Health, University of Michigan, Ann Arbor, MI 48109, USA; cookstev@umich.edu (S.C.); nancyfl@umich.edu (N.L.F.); rmeza@umich.edu (R.M.); janahirs@umich.edu (J.L.H.); 4 Department of Health Behavior and Health Education, School of Public Health, University of Michigan, Ann Arbor, MI 48109, USA; riteshm@umich.edu

**Keywords:** umbrella review, health effects, electronic cigarettes, vaping, tobacco products

## Abstract

E-cigarettes are often marketed as a safer alternative to combustible cigarettes. However, their health effects, especially those associated with long-term use, remain largely uncertain. We conducted an umbrella review of the cardiopulmonary and carcinogenic risks of e-cigarette use, distinguishing between short-term and long-term health effects. The search for systematic reviews was conducted across four electronic databases through 25 January 2022. Methodological quality was assessed using the AMSTAR-2 quality appraisal tool. Seventeen systematic reviews, including five meta-analyses, were included in our umbrella review. There was a clear underreporting of e-cigarette devices and e-liquid types, e-cigarette and cigarette exposure, and the health and smoking status of study participants. Overall, the findings suggest that short-term use of e-cigarettes may be associated with acute cardiopulmonary risks, although to a lesser extent than cigarette use. Long-term e-cigarette use may have pulmonary/respiratory benefits in those who switch from chronic cigarette smoking, particularly in individuals with asthma and chronic obstructive pulmonary disease (COPD). Evidence on intermediate and long-term carcinogenic effects is lacking. This umbrella review underscores the urgent need for systematic reviews with better adherence to established reporting guidelines, consistent definitions of duration of e-cigarette use, a focus on newer devices, and accounting for the impacts of former or current smoking.

## 1. Introduction

The electronic cigarette (EC) is a novel nicotine delivery system designed to produce an aerosol by heating a liquid that usually contains nicotine along with other additives. Unlike traditional cigarettes (TCs), ECs do not combust tobacco and do not produce “tar” or carbon monoxide, two of the most harmful elements in tobacco smoke [1]. Despite often being marketed as a safer alternative to combustible tobacco products, the harms of ECs, especially those associated with long-term use, remain largely uncertain. In addition, the potential health benefits of EC use relative to smoking are not very clear. A substantial amount of research has been conducted in the past decade to examine the health effects of EC use, often providing conflicting evidence and claims [2,3,4,5].

Many reviews have attempted to summarize the evidence by assessing studies that often vary in their methods, the population considered, and the health outcome measures used. The present work aims to evaluate and summarize the findings from the existing systematic reviews by conducting an umbrella review. To our knowledge, only one umbrella review of EC health effects has been conducted to date [6]. It focused on cardiovascular health risks but, importantly, did not distinguish those risks by TC smoking status. We expand on this work by (a) including pulmonary, respiratory, and carcinogenic risks; (b) distinguishing between short-term and long-term health effects; (c) considering specific populations (e.g., people with asthma or chronic obstructive pulmonary disease (COPD)); and (d) considering both the absolute harm of ECs and the harm of ECs relative to TC use.

Since most EC users are dual users or former smokers [7] and many tobacco-associated health risks decline gradually following smoking cessation [8], this study aims to address whether reviews on health effects of EC use differentiate these effects between tobacco-naïve EC users, EC-naïve smokers, dual EC and TC users, and former smokers who switched to EC use. Finally, we examine the methodological limitations of the existing systematic reviews and provide recommendations for future research.

## 2. Materials and Methods

The peer-reviewed umbrella review protocol was developed a priori in adherence with the guidelines for umbrella reviews [9] and registered with PROSPERO (Registration Number: CRD42021237878). Initially, the protocol also planned to include evidence on the chemical constituents of ECs and their toxic health effects. However, due to the large volume of identified literature, we evaluated these outcomes in a separate umbrella review. We conducted the umbrella review in adherence with the Preferred Reporting Items for Systematic reviews and Meta-analyses (PRISMA) [10].

### 2.1. Search Strategy

Two reviewers (CJC and NT) designed and performed a structured literature search in PubMed, Web of Science, Embase, and Cochrane Database of Systematic Reviews with no restrictions on the publication date. The following search terms were included: “electronic cigarette”, “e-cigarette”, “electronic nicotine delivery system”, “personal vaporiser”, “personal vaporizer”, “e-liquid”, “nicotine content”, “systematic review”, “meta-analysis” and “review”. Gray literature searches were conducted in OpenGrey and Google custom search limited to U.S. government and non-profit organization domains (i.e., gov, .org). The initial search was conducted on 27 May 2020, and was updated on 25 January 2022. The complete search strategy employed for PubMed is presented in Supplementary File S1.

Two reviewers (NT and CJC in the initial search; and NT and MK in the updated search) independently extracted and screened titles and abstracts. The reviewers were blinded to author names and affiliations as well as the journals of publication. Two reviewers (NT and MK) independently screened the full-text articles for eligibility criteria. If the full-text articles were not accessible, the first authors were contacted with inquiries. Finally, we searched reference lists in the bibliographies of eligible papers for additional sources. Any disagreements between reviewers were discussed and resolved either by consensus or by consulting a third reviewer (DTL).

### 2.2. Eligibility

This review included systematic reviews and meta-analyses published in English that investigated any of three outcome categories: (1) cardiovascular effects, (2) respiratory or pulmonary effects, and (3) carcinogenic effects. A literature review was considered systematic if it explicitly identified itself as a systematic review in the title, abstract, keyword, or methods and was peer-reviewed. 

Systematic reviews were included if they examined at least one outcome of interest. Evidence from in vitro, animal, and human studies was included. Human studies included healthy adult participants and adults with underlying cardiovascular, pulmonary, or oncological conditions (self-reported or physician-diagnosed) to assess the potential effects on disease improvement or progression. There was no limitation on the types of EC devices and e-liquids considered.

Since our focus was on the clinical/laboratory-measured physiological and pathophysiological health effects of ECs, self-reported adverse events (e.g., dry cough, throat irritation, and anxiety) were excluded. As our study focused on health outcomes from the intended use of ECs, we excluded reviews on health effects resulting from the misuse of ECs, such as poisoning, or outcomes associated with vaping illicit substances, as occurred in the instance of the e-cigarette or vaping product use-associated lung injury (EVALI). Safety outcomes related to device malfunction, such as burns and injuries, were also excluded. Finally, because we focused on direct health effects, we excluded reviews on the health effects of passive vaping.

### 2.3. Data Extraction

A standardized data extraction tool was developed and employed independently by two reviewers (NT and MK) to extract the following information from each included review: citation, author financial disclosures, review objectives, details of the literature search, designs of included studies, description of exposure (e.g., duration of use, EC device, and e-liquid types), health outcome variables, sample types (for preclinical studies), details on the population smoking status and history of tobacco use (for human outcomes), quality appraisal tool used, main results, authors’ conclusions along with recommendations, and study limitations. When extracting evidence, we attempted to differentiate between acute and long-term effects as defined by the authors of the included reviews.

### 2.4. Assessment of Quality and Risk of Bias

Reviews that were eligible for inclusion were appraised for quality using the AMSTAR-2 checklist [11]. While several other quality appraisal tools for systematic reviews exist, AMSTAR-2 is the most commonly used tool to evaluate the methodological quality of systematic reviews (with or without a meta-analysis) of both randomized and non-randomized studies [12,13]. The checklist is comprised of 16 questions and includes seven domains that can critically influence the validity of a review and its conclusions. Critical domains include an a priori registered review protocol, adequacy of the literature search, justification for the exclusion of individual studies, risk of bias assessment, appropriate statistical methods for combining results and investigating publication bias (for meta-analysis), and consideration of potential biases when interpreting the results of individual studies [11].

Given the heterogeneity in the reviews and meta-analyses we included, and concerns about the potentially limited discriminative power of the tool due to the previously observed abundance of critically low ratings [14], the overall confidence score for systematic reviews was not derived. Instead, we address the limitations of each systematic review based on the individual items of the tool. Two reviewers (NT and MK) independently performed the assessment. Any disagreements were discussed and resolved by consensus.

## 3. Results

**Figure 1:**
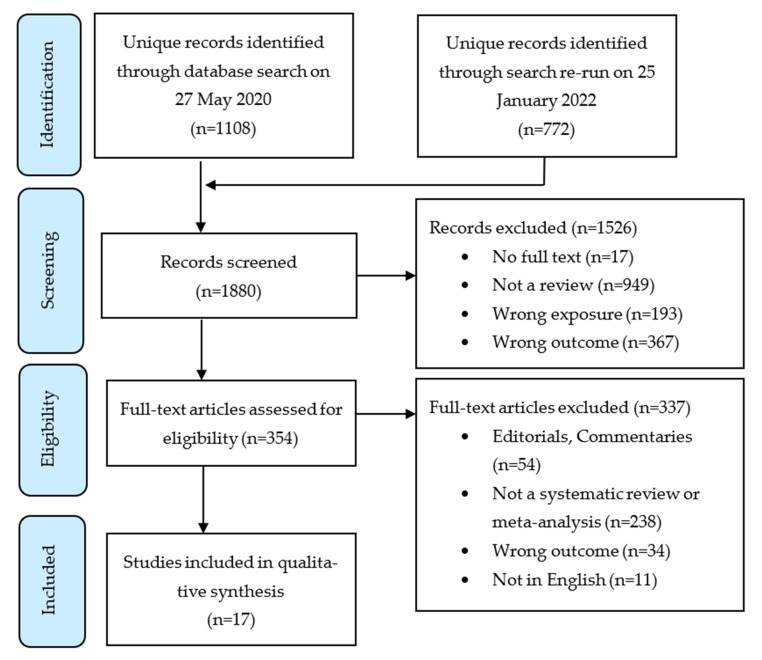
Flow diagram of the selection process for the umbrella review.

Our literature search identified 1880 unique records. The titles and abstracts were screened for eligibility, excluding 1526 records. Full-text articles were accessed for the remaining 354 records and thoroughly assessed for exclusion criteria (Figure 1).

The most common reason for excluding full texts was the unfulfilled criterion of being a systematic review or meta-analysis (n = 238). Potentially relevant literature reviews that were excluded as non-systematic are presented in Supplementary Table S1. Seventeen systematic reviews, including five meta-analyses, were ultimately included in our study. Eleven reviews summarized evidence on cardiovascular effects, thirteen on pulmonary/respiratory effects, and five on carcinogenic effects of ECs. An overview of the health domains included in systematic reviews is presented in Supplementary Table S2. Information on the studied EC device types was provided in eight reviews, and on e-liquid type (e.g., containing nicotine) in seven reviews. Among reviews on human health effects, the smoking status of the studied individuals was reported in ten, and the health status was reported in seven reviews. Table 1, Table 2 and Table 3 summarize the main characteristics of the included reviews and meta-analyses.

### 3.1. AMSTAR 2 Assessment of Quality and Risk of Bias

The quality assessment of the included reviews is presented in Table 4. Thirteen [15,16,17,18,19,20,21,22,23,24,25,26,27] of the seventeen reviews failed to report whether their methods were established in a written protocol prior to conducting their review. Two [28,29] published a peer-reviewed protocol but did not provide sufficient details or justifications for deviations from the protocol, and one [30] briefly mentioned a review protocol without any details on its peer-review or registration status.

Nine [18,19,20,22,23,25,26,27,28] reviews failed to apply a comprehensive literature search strategy, and all but two [15,24] reviews failed to provide a list of excluded studies. An appropriate technique for the systematic assessment of the quality and risk of bias of included studies was applied only in eight [15,16,17,22,24,25,29,31] reviews. Twelve [15,16,17,21,22,23,24,25,26,29,30,31] reviews discussed heterogeneity in their results, and only seven [15,16,17,18,21,27,30] reported any conflict of interest/funding received by the authors of the included studies.

All five [22,23,25,29,31] meta-analyses used appropriate methods for the statistical combination of results. One [23] failed to apply a satisfactory technique to assess the quality and risk of bias of the included studies, while two [22,25] did not assess the potential impact of the risk of bias in individual studies on the overall findings. The risk of publication bias was assessed in four [23,25,29,31] meta-analyses.

**Table 1:** Characteristics of systematic reviews on the cardiovascular health risks of e-cigarettes.

Author, Year	Funding/COI Disclosures of Review Authors	Review Objectives	Date Range of Literature Search	Evidence Type	Health Outcome Measures		Study Designs	Population	Number of Included Studies	Exposure	Vaping Devices
					Acute	Chronic					
**Garcia et al., 2020 [30]**	The authors declare no conflict of interest. The work was supported by the Tobacco-Related Disease Research Program (TRDRP)	Synthesize studies that have investigated the autonomic CV effects of ECs in humans: (1) Acute effects of ECs vs. TCs. (2) Comparison of acute effects by nicotine vs. non-nicotine containing ECs. (3) Chronic Effects of ECs (in non-TC smokers). (4) Relative chronic effects of ECs compared to TCs (switching).	Through December 2019	Human	Acute (minutes to hours after EC use) changes in heart rate variability (HRV), heart rate (HR), systolic blood pressure (SBP), and diastolic blood pressure (DBP).	Chronic * (days or longer) changes in HRV, HR, SBP, and DBP	Experimental (control trials)	Aim 1: TC smokers. Aim 2: TC smokers and nicotine-naive participants. Aim 3: chronic EC users (no TC or dual use). Aim 4: chronic TC smokers.	19 in total. Eight on acute effects of TC versus EC (with or without nicotine). Five on acute effects of EC with vs. without nicotine. Two on chronic effects of EC. Six on switching from chronic TC to chronic EC use.	Acute and chronic EC use (with or without nicotine).	Earlier generation EC devices
**Skotsimara et al., 2019 § [22]**	The authors declare no conflict of interest.	Meta-analysis and systematic review of studies that have investigated: 1. Acute effects of ECs. 2. Effects of switching from TC use to chronic EC use on a CV system.	January 2000–November 2017	Human, in vitro	Acute (5–30 min after EC use) changes in HR, SBP, and DBP	Chronic * (assessment time range 5 days–1 year) changes in HR, SBP, and DBP. Secondary outcomes: Arterial stiffness, endothelial function, myocardial function, and risk of cardiovascular events.	Experimental (randomized and non-randomized control trials) for meta-analysis. Experimental bench studies and clinical trials for Systematic Review.	Studies included healthy smokers (10), hypertensive smokers (1), healthy non-smokers (1) and EC users (or dual users) (2).	26 studies included in a systematic review, 14 studies in the meta-analysis. Of 14, 11 studied acute effects (of them 11 on HR and 7 on BP); three studied chronic effects.	Acute and chronic EC use. Duration of exposure in in vitro studies not reported.	In meta-analysis, a classic tobacco EC in rechargeable cartomizer 2.4% nicotine, 75% glycerin vehicle was the commonest type used across studies. When different nicotine EC types were used, the higher nicotine type was included. Where relevant, only non-flavored EC type was used.
**Kennedy et al., 2019 [15]**	N/A	Summarize of physiological and pathophysiological cardiovascular effects after direct exposure to EC.	1996 to June 2019	Human, Animal, in vitro	Sympathetic nerve activation (HR, HRV, SBP, and DBP), oxidative stress, endothelial function, and platelet activation.	N/A	Experimental (randomized control trials, non-randomized control trials, randomized crossover studies, and non-randomized crossover studies).	Adults with or without cardiovascular disease, independent of smoking status or age.	38 studies total. Of them, 24 human studies (18 measured HR, 17 measured BP); six animal studies, and eight cardiovascular cell culture studies.	Direct EC use (human studies), Inhalation of EC vapor (animal studies); Cellular exposure to EC vapor (range 4 to 72 h).	Devices varied across studies, predominantly first and second generation EC devices.
**Glasser et al., 2017 [28]**	N/A	Synthesize empirical studies on electronic nicotine delivery systems across a broad range of topics, including the health effects.	Through 31 May 2016	Human, in vitro	Physiologic health effects, such as HR and blood pressure (time of assessment unspecified). Anti-inflammatory process, oxidative stress, and changes in cell apoptosis.		Experimental, quasi-experimental, observational (case control, cohort, and cross-sectional studies), case reports, case series, qualitative studies, mixed methods, and in vitro.	N/A	129: 116 articles on the impact of vaping on human health and 13 on animal health.	Exposure to vapor (duration not specified).	N/A
**Pisinger & Dossing, 2014 [21]**	The authors declare no conflict of interest.	(1) Systematic and critical review of the existing literature on the health consequences of ECs and discuss the implications of our findings for public health; (2) to investigate how many of the published articles have a conflict of interest.	Through 24 August 2014	Human	HR, blood pressure, oxygen saturation, and cardiac function.	N/A	Experimental	Not specified.	Eight studies on cardiovascular health effects.	Short-term EC use (minutes).	N/A
**Bozier et al., 2020 [18]**	The authors declare no conflict of interest.	(1) To provide a comprehensive update of data on the potential health effects of ECs since the NASEM report. (2) To provide a focused discussion of the scientific literature that will help inform the general public, health-care practitioners, and policy makers of the effects of EC use on health.	February 2017 through May 2019	Human, Animal	HR, HRV, blood pressure, and arterial stiffness (time of assessment unspecified).		Experimental, observational, and case reports.	Not specified.	Eight studies on cardiovascular health effects.	EC use and exposure to EC vapor (no data on the duration).	N/A
**Harrell et al., 2014 [19]**	The authors declare no conflict of interest. One co-author reports to be receiving research support from a pharmaceutical company, a manufacturer of a stop smoking medication.	To have a summary of the current, relevant literature on EC safety and efficacy.	Through November 2013	Human	Acute changes in HR, inflammatory markers, myocardial function, and arrhythmia (no definition of “acute”).	N/A	Case series and case reports.	Not specified. The results show that some studies included smokers and never smokers. No information on their health.	Eight studies on acute physiological effects.	EC use (no data on the duration).	EC devices reported partially in a Table with study characteristics. No summary or discussion provided.
**Ioakeimidis et al., 2016 [20]**	N/A	To highlight the efficacy for smoking cessation and the potential hazards of EC use.	Through June 2015	Human	Acute and long-term cardiovascular outcomes (SBP, DPB, arrhythmia, aortic stiffness, and myocardial relaxation) (No definition of “acute” or “long-term”).		Not specified. Original studies as well as review articles and statements are included.	N/A	20 original studies and eight review articles and statements.	EC use (no data on the duration).	N/A
**NASEM, 2018 [16]**	N/A	To evaluate the available evidence of the health effects related to the use of electronic nicotine delivery systems (ENDS) and identify future federally funded research needs.	1 February 2017–31 August 2017	Human	Acute CV outcomes: HR, SBP, DBP, oxidative stress, endothelial function, arterial stiffness, and cardiac geometry and function.	Long-term CV outcomes: clinical (coronary heart disease, stroke, and peripheral artery disease) and subclinical atherosclerosis (carotid intima-media thickness, and coronary artery calcification).	Experimental, observational	In acute effects studies: a range of 23 to 39 y.o. In longer-term effect studies: a range of 33 to 54 y.o Generally healthy participants, one study included participants with hypertension.	13 studies on acute and three studies on longer-term cardiovascular effects.	Acute (minutes to hours) and long-term (not further defined) vaping.	A tank-style device in one study; second-generation devices in three studies; cigalikes in six studies; one leading brand of an unspecified device in one study; and the personal devices of the study participants in two studies.
**Goniewicz et al., 2020 [24]**	First author received research grant from Pfizer and personal fees from Johnson & Johnson outside of this work. The other authors had nothing to declare.	To conduct a systematic review of epidemiological studies that estimated the odds of key cardiovascular outcomes among EC users who formerly smoked, compared with current smokers who do not use ECs.	Through September 2020	Human	Cardiovascular outcomes (e.g., stroke, myocardial infarction, coronary heart disease).		Observational	Adult former smokers who transitioned to EC use and current exclusive TC smokers (as a comparator).	Three cross-sectional studies.	EC use (no data on the duration).	N/A
**Larue et al., 2021 § [29]**	No competing interests for this work were declared. The review protocol declares that a family member of the lead author works in tobacco industry.	To assess the immediate cardiovascular effects of acute EC use.	Through 20 May 2021	Human	SBP, DBP, HR	N/A	Experimental	Mostly healthy participants. Current smokers and non-smokers.	22 cross-over and randomized trial studies.	Duration of EC use (with and without nicotine) ranged from 3 to 30 min. Assessment time occurred between 1 and 30 min post use.	Different brands of ECs with varying nicotine content.
**Martinez-Morata et al., 2020 [26]**	The authors declare no conflict of interest.	To summarize the available studies on the short-term effects of ECs on blood pressure in experimental studies, and the association between ECs and blood pressure endpoints in observational studies.	2003 through April 2020	Human	SBP, DBP	N/A	Experimental, observational	Healthy adult participants with no prior diagnosis of hypertension.	13 randomized trials and one prospective study.	EC use with outcome assessment up to 4 h post use.	Information was collected and presented in a tabular format. No stratified analysis by device type or discussion of the impact of device type on the outcome was provided.

§ Meta-analysis. COI = conflict of interest; EC = electronic cigarettes; TC = traditional cigarettes; SBP = systolic blood pressure; DBP = diastolic blood pressure; HR = heart rate; HRV = heart rate variation; and N/A = not available. * Chronic effects were described as those measured in chronic EC users (non-TC smokers) and in TC smokers after switching to chronic EC.

**Table 2:** Characteristics of systematic reviews on respiratory/pulmonary health risks of e-cigarettes.

Author, Year	Funding/COI Disclosures of Review Authors	Objectives	Date Range of Literature Search	Evidence Type	Health Outcome Measures		Study Designs	Population	Number of Included Studies	Exposure	Vaping Devices
					Acute	Chronic					
**Glasser et al., 2017 [28]**	N/A	Synthesize empirical studies on electronic nicotine delivery systems across a broad range of topics, including the health effects.	Through 31 May 2016	Human	Lung function, changes in COPD/asthma disease symptoms (time of assessment unspecified).		Experimental, quasi-experimental, observational (including case control, cohort and cross-sectional), case reports, case series, qualitative studies, mixed methods.	N/A	116 articles on the impact of vaping on human health.	Exposure to vapor (duration not specified).	N/A
**Gualano et al., 2014 [17]**	N/A	A systematic review of published studies in order to investigate the efficacy and the adverse effects of the EC.	Through April 2014	Human	Respiratory resistance	N/A	Experimental	N/A	One study	Short-term use of EC (duration not specified).	N/A
**Pisinger & Dossing, 2014 [21]**	Authors report no conflict of interest.	(1) Systematic and critical review of the existing literature on the health consequences of ECs and discuss the implications of our findings for public health; and (2) to investigate how many of the published articles have a conflict of interest.	Through 24 August 2014	Human, Animal	Acute changes in airway resistance, obstruction, and spirometry-assessed lung function.	N/A	Experimental	Human studies: Generally healthy participants. The results show that three studies included participants with asthma/COPD. Animal: mice.	Six human and one animal studies on pulmonary health effects.	Short-term EC use (minutes) * Intra-tracheal application of a diluted e-liquid solution 2× week for 2 weeks.	N/A
**Bozier et al., 2020 [18]**	None declared	(1) To provide a comprehensive update of data on the potential health effects of ECs since the NASEM report. (2) To provide a focused discussion of the scientific literature that will help inform the general public, health-care practitioners, and policy makers of the effects of EC use on health.	February 2017 through May 2019	Human, Animal	Changes in lung function in animals and humans. Changes in respiratory symptoms in COPD/asthma patients.		Experimental, observational, and case reports.	Not specified.	14 studies on respiratory/pulmonary health effects.	Short-term and long-term EC use or exposure to EC vapor (no data on the duration).	N/A
**Harrell et al., 2014 [19]**	The authors report no conflicts of interests. One co-author reports to be receiving research support from a pharmaceutical company, which is also a manufacturer of a stop smoking medication.	To have a summary of the current, relevant literature on EC safety and efficacy.	Through November 2013	Human	Acute changes in respiratory resistance, lung function (no definition of “acute”).	N/A	Case series and case reports.	Not specified. The results show that some studies included smokers and never smokers. No information on their health.	Eight studies on acute physiological effects.	EC use (no data on the duration).	EC devices reported partially in a Table with study characteristic. No summary or discussion provided.
**NASEM, 2018 [16]**	N/A	To evaluate the available evidence of the health effects related to the use of electronic nicotine delivery systems (ENDS) and identify future federally funded research needs.	1 February 2017–31 August 2017	Human	Intermediate outcomes (measurements of lung function and lung structure, quantification of inflammatory cell numbers from bronchoalveolar lavage (BAL), pro-inflammatory cytokines from bronchial biopsies, and improvement and progression of existing respiratory diseases.	Development of respiratory conditions, such as asthma, pneumonia, and COPD.	Experimental, observational.	Subjects with or without preexisting respiratory disease.	17 human studies.	Acute (minutes to hours) and long-term (not further defined) vaping.	
**Ioakeimidis et al., 2016 [20]**	N/A	To highlight the efficacy for smoking cessation and the potential hazards of EC use.	Through June 2015	Human	Acute effects on pulmonary function (No definition of “acute”).	N/A	Not specified. Original studies as well as review articles and statements.	N/A	20 original studies and 8 review articles and statements	EC use (no data on the duration)	N/A
**Bravo-Gutierrez et al., 2021 [27]**	Authors declared no conflicts of interest.	To describe the adverse effects on the respiratory system in consumers of ECs.	January 2013–August 2020	Human, animal, in vitro	Acute and sub-acute effects on DNA damage, inflammation mechanisms, and reactive oxygen species (ROS) presence.	Chronic effects on DNA damage, inflammation mechanisms, ROS presence.	Experimental, laboratory.	N/A	N/A	Acute (2 h daily for 3 days), sub-acute (2 h daily, 5 days a week for 30 days), and chronic (no definition) inhalation	N/A
**Chand & Hosseinzadeh, 2021 § [31]**	Authors declared no conflicts of interest.	To evaluate the most recent studies exploring the association between EC use and asthma worldwide.	2007–March 2021	Human	Self-reported asthma diagnosis.		Observational	Youth and adult current EC users, ever EC users and dual EC and TC users.	13 cross-sectional studies.	Current EC use (past 30 days), ever EC use (ever use but not in past 30 days), dual use (use of EC and TC in past 30 days).	N/A
**Larue et al., 2021 § [29]**	No competing interests for this work were declared. The review protocol declares that a family member of the lead author works in tobacco industry.	To assess the immediate respiratory effects of acute EC use.	Through 20 May 2021	Human	Augmentation index, fraction of exhaled nitric oxide (FeNO), and spirometry measures.	N/A	Experimental	Mostly healthy participants. Current smokers and non-smokers.	17 cross over and randomized trial studies.	Duration of EC use (with and without nicotine) ranged from 3 to 30 min. Assessment time occurred between 1 and 30 min post use.	Different brands of ECs with varying nicotine content.
**Xian & Chen, 2021 § [25]**	Authors declared no conflicts of interest.	To explore the relationship between ECs and the risk of asthma.	Through August 2020	Human	Asthma diagnosis		Observational	Adolescents and adults with diagnosed asthma, who are current or former EC users or smokers (comparators).	11 cross-sectional studies.	Current and former EC use (no further definition).	N/A
**Goniewicz et al., 2020 [24]**	First author received research grant from Pfizer and personal fees from Johnson & Johnson outside of this work. Other authors have nothing to declare.	To conduct a systematic review of epidemiological studies that estimated the odds of key respiratory outcomes among EC users who formerly smoked, compared with current smokers who do not use ECs.	Through September 2020	Human	Respiratory outcomes (COPD, chronic bronchitis, emphysema, asthma, and wheezing).		Observational	Adult former smokers who transitioned to EC use and current exclusive TC smokers (as a comparator).	Two cross-sectional and one prospective.	EC use (no data on the duration).	N/A
**Wills et al., 2021 § [23]**	Authors declared no conflicts of interest.	To provide a comprehensive review and meta-analysis of evidence from epidemiological studies about the association of EC use with asthma and COPD in human populations.	Through March 2020	Human	Asthma and COPD.		Observational	General population of adolescents and adults.	15 studies on asthma and nine for COPD (seven cross-sectional and two prospective).	EC use (current use, 30-day use, and long-term use).	N/A

§ Meta-analysis. COI = conflict of interest; EC = electronic cigarettes; TC = traditional cigarettes; COPD = chronic obstructive pulmonary disease; ROS = reactive oxygen species; FeNo = fraction of exhaled nitric oxide; and N/A = not available. * Duration of exposure varied according to the followed intervention protocols.

**Table 3:** Characteristics of systematic reviews on carcinogenic risks of e-cigarettes.

					Acute	Chronic					
**Pisinger & Dossing, 2014 [21]**	Authors report no conflict of interest.	(1) Systematic and critical review of the existing literature on the health consequences of ECs and discuss the implications of our findings for public health. (2) To investigate how many of the published articles have a conflict of interest.	Through 24 August 2014	Human	Potentially carcinogenic product in human biological samples,	N/A	Experimental	Most studies included smokers,	One	N/A	N/A
**Bozier et al., 2020 [18]**	None declared	(1) To provide a comprehensive update of data on the potential health effects of ECs since the NASEM report. (2) To provide a focused discussion of the scientific literature that will help inform the general public, health-care practitioners, and policy makers of the effects of EC use on health.	February 2017 through May 2019	Human	Potentially carcinogenic compounds breath and in respiratory tract retention.	Potentially carcinogenic compounds in urine.	Experimental, observational, and case reports.	EC users. Non-smoking chronic users.	Two	Short-term use (no data on the duration) and long-term use (≥6 months).	N/A
**Harrell et al., 2014 [19]**	The authors report no conflicts of interests. One co-author reports to be receiving research support from a pharmaceutical company, which is also a manufacturer of a stop smoking medication.	To have a summary of the current, relevant literature on EC safety and efficacy.	Through November 2013	In vitro	Gene mutations (not specified whether acute or long-term).		Preclinical	Bronchial epithelial cells.	One	Exposure to EC vapor (no data on the duration).	N/A
**NASEM, 2018 [16]**	N/A	To evaluate the available evidence of the health effects related to the use of electronic nicotine delivery systems (ENDS) and identify future federally funded research needs.	Between 1 February 2017, and 31 August 2017	Human, animal, and in vitro	DNA damage, intermediate cancer endpoints (biomarkers).	N/A	Preclinical, observational, case reports.		Four humans, three in vitro studies.	Acute (minutes to hours) EC use or vapor exposure.	N/A
**Ioakeimidis et al., 2016 [20]**	N/A	To highlight the efficacy for smoking cessation and the potential hazards of EC use.	Through June 2015	In vitro	Changes in gene expression.	N/A	Not specified. Original studies as well as review articles and statements are included.	Bronchial cells	20 original studies and eight review articles and statements.	EC use (no data on the duration).	N/A

COI = conflict of interest; EC = electronic cigarettes; TC = traditional cigarettes; and N/A = not available.

**Table 4:** Quality appraisal of the included systematic reviews using the AMSTAR-2 tool.

	Research Question Included PICO	* Review Methods Established Prior to Review	Explanation of Study Design Selection	* Comprehensive Literature Search Strategy	Study Selection Performed in Duplicate	Data Extraction Performed in Duplicate	* List of Excluded Studies with Justification	Describe Studies in Adequate Detail	* Satisfactory Technique for Assessing RoB		Report Sources of Funding	* Use Appropriate Methods for Statistical Combination of Results (for Meta-Analysis)	Assess Potential Impact of RoB in Individual Studies on the Results (for Meta-Analysis)	* Account for RoB in Individual Studies when Interpreting Results	Explanation for and Discussion of Heterogeneity in Results	* Investigate publication Bias and Discuss Impact (for Quantitative Synthesis)	Reported any Conflict of Interest
									RCT	NRSI							
Kennedy et al., 2019 [15]										NA		NA	NA			NA	
NASEM, 2018 [16]												NA	NA			NA	
Gualano et al., 2014 [17]												NA	NA			NA	
Bozier et al., 2020 [18]												NA	NA			NA	
Garcia et al., 2020 [30]												NA	NA			NA	
Glasser et al., 2017 [28]												NA	NA			NA	
Harrell et al., 2014 [19]												NA	NA			NA	
Ioakeimidis et al., 2016 [20]												NA	NA			NA	
Pissinger & Dossing 2014 [21]									NA			NA	NA			NA	
Skotsimara et al., 2019 § [22]									NA								
Wills et al., 2021 § [23]									NA								
Goniewicz et al., 2020 [24]									NA			NA	NA			NA	
Xian & Chen, 2021 § [25]									NA								
Martinez-Morata et al., 2021 [26]												NA	NA			NA	
Larue et al., 2021 § [29]										NA							
Chand & Hosseinzadeh, 2021 § [31]									NA								
Bravo-Gutierrez et al., 2021 [27]												NA	NA			NA	

* Critical domains. § Meta-analysis. Green = yes; red = no; and orange = partial yes. PICO = Population or participants, and conditions of interest; Interventions or exposures; Comparisons or control groups; Outcomes of interest; RCT = randomized controlled trials; NRSI = non-randomized studies of interventions; RoB = risk of bias; and NA = Not applicable.

### 3.2. Cardiovascular Health Effects

Twelve systematic reviews, including two meta-analyses, reported evidence on acute [15,16,19,20,21,22,26,29,30] and long-term [16,20,22,24,30] cardiovascular effects of EC use, or on the effects of unspecified duration of EC use [18,28]. Acute cardiovascular outcomes were more frequently reported and usually included those measured following short-term EC use (minutes to hours) [16,22,26,29,30]. In several reviews [15,19,20,21], the duration of a short-term EC use was not further defined. The effects of long-term exposure (spanning several days to several years [22,30], although sometimes unspecified) [16,20,24] were much less frequently reported.

#### 3.2.1. Human Studies

The most commonly reported short term outcomes were changes in heart rate (HR) [15,16,18,19,20,22,29,30], heart rate variability (HRV) [15,16,30], and in systolic (SBP) and diastolic blood pressure (DBP) [15,16,18,20,22,26,29,30]. Other acute outcomes included oxygen saturation [21], inflammatory markers [19], arterial stiffness [16,19], endothelial function [16,29], oxidative stress [16,29], and cardiac geometry and function [16].

Long-term outcomes included changes in HR [16,22,30], HRV [16,30], SBP and DBP [16,22,30], biomarkers of inflammation and oxidative stress [16], risk of cardiovascular events [22,24], as well as subclinical outcomes, such as vascular/arterial stiffness [22], endothelial and myocardial functions [22]. A detailed overview of the results, conclusions, and recommendations of systematic reviews by outcome category are presented in Supplementary Table S3.

##### Acute Effects

The report of the National Academies of Science, Engineering and Medicine (NASEM) [16] found, based on their strength of evidence framework for conclusions, that there is substantial evidence of an associated between acute nicotine-containing EC use and increases in HR, DBP, and limited evidence (i.e., supportive findings from fair quality studies or mixed findings with most favoring one conclusion) of an association with the increase in SBP. Further, the report found limited evidence of increases in endothelial dysfunction, arterial stiffness, or biomarkers of oxidative stress.

Two reviews [15,30] reported increased HR, SBP, and DBP in participants after nicotine-containing EC use. However, one [15] did not stratify these findings by participants’ TC smoking status. One meta-analysis [22] found an increase in HR, SBP, and DBP after the use of nicotine ECs; however, the effects were not examined by study participants’ TC smoking status. In another meta-analysis [29], the use of nicotine ECs was associated with increases in HR, SPB, and DBP, comparable to the effects seen after TC use, with no significant changes observed after nicotine-free EC use.

In contrast, one review found that the use of ECs without nicotine also resulted in increased SBP and DBP, although less compared to nicotine containing ECs [26]. Other reviews similarly reported acute increases in HR [19,20,30], SBP [20,30], and DBP [20,30] after EC use (unknown nicotine content) but of smaller magnitude than TC use. There were indications of worse endothelial function and greater oxidative stress [29] and no significant immediate effect on myocardial function compared to TC use [19,20].

##### Long-Term Effects

The NASEM report [16] found insufficient evidence that chronic EC use is associated with long-term changes in HR, blood pressure, and cardiac geometry and function. Another review [22] reported that the adjusted risk of myocardial infarction was increased in EC users compared to non-users, although to a lesser extent than in TC smokers. However, the authors noted that these findings were sensitive to non-random misclassification bias regarding EC users and smokers with prior myocardial infarction diagnosis.

##### Effects of EC Use by Smoking Status

- Tobacco-naïve EC users

Short-term use of both nicotine and nicotine-free ECs elevated SBP and DBP in non-smokers [26]. In long-term (days or longer) exclusive nicotine EC users, only HRV, and not blood pressure or HR, was chronically elevated compared to non-users [30].

- Former TC smokers who switch to ECs

The NASEM report [16] indicated substantial evidence that completely switching from regular use of TCs to ECs reduces the short-term adverse health outcomes in several organ systems. Other reviews suggested that switching from TC use to chronic EC use may reduce cardiovascular harm [18] and regulate blood pressure [22,30] with no effect on HR [22,30]. In contrast, one review [24] found no difference in the risk of myocardial infarction or coronary heart disease for former smokers who transitioned to ECs compared to current exclusive TC smokers.

- EC-naïve current smokers

Short-term EC use with and without nicotine was associated with acute elevations of HR [21], SBP [26], and DBP [21,26] in EC-naïve TC smokers.

- Dual (EC and TC) users

The NASEM report [16] found no evidence that long-term dual EC and TC use changed morbidity or mortality compared with exclusive TC use. One review [18] suggested a possible increased risk of cardiovascular disease for dual users of TCs and ECs compared to the exclusive use of either product.

#### 3.2.2. In Vivo Animal Studies

Murine models exposed to EC vapor displayed vascular inflammatory markers and angiogenesis as well as increased development of atherosclerotic plaque, suggestive of endothelial dysfunction and increased atherosclerotic risk [15]. Some physiological effects (i.e., increased oxidative stress, elevated blood pressure, and decreased HR) were reported in animal models after EC vapor exposure but much less than from TC exposure [18,28].

#### 3.2.3. In Vitro Cell Studies

One review [15] suggested an increase in reactive oxygen species (ROS) production, a decrease in antioxidant levels, and disorder in endothelial cellular culture function and interactions after short-term (4–72 h) exposure of cardiovascular cell cultures to EC aerosol extract. The NASEM report [16] similarly stated that there was substantial evidence that EC aerosols can induce acute endothelial cell dysfunction and increase oxidative stress. However, these effects were generally lower than from TC smoke. Other reviews found no gene expression alterations in human coronary artery endothelial cells [22] but reported increases in anti-inflammatory processes, oxidative stress, cell apoptosis and necrosis [28] after exposure to EC aerosol.

### 3.3. Respiratory/Pulmonary Health Effects

The effects of EC use on pulmonary and respiratory health were reported in thirteen systematic reviews, including four meta-analyses. Seven reviews examined acute effects (minutes to hours post EC exposure but often undefined) [16,17,19,20,21,27,29], one examined sub-acute effects (two hours a day, five days a week for 30 days) [27], and two considered chronic effects (not further defined) [16,27]. Six reviews explored the association between EC use status and prevalence of chronic respiratory conditions [18,23,24,25,28,31].

#### 3.3.1. Human Studies

Acute outcomes most commonly included spirometry-assessed pulmonary function [16,19,21,29], respiratory resistance [17], and fraction of exhaled nitric oxide (FeNO) [20,29]. Chronic outcomes included diagnosed asthma [16,23,24,25,31], chronic bronchitis [24], emphysema [24], and COPD [16,23,24].

##### Acute Effects

The NASEM report [16] found that ECs containing nicotine, but not nicotine-free ECs, have short-term effects on the lung defense mechanism. Two reviews reported a decrease in FeNO [20,29] in response to EC use, while a meta-analysis [29] found that the decrease is likely related to the nicotine content. Other reviews reported an airway obstruction comparable to smoking, with a smaller impact on lung function [21]; and reduced lung function, especially from nicotine-containing ECs, although less than from TC smoke [19]. In contrast, a meta-analysis found no significant changes in spirometry measures following EC use, regardless of the nicotine content [29]. An increased respiratory resistance [17], upper respiratory tract irritation, and allergic airway inflammation [20] were also reported following EC use.

##### Effects in Individuals with COPD and Asthma

The NASEM report [16] indicated limited evidence of improved lung function and respiratory symptoms among people with asthma and of a reduction in exacerbations in COPD patients after switching completely or partly (dual use) from smoking to ECs. The report also found moderate evidence for EC use and increased asthma exacerbations. One review [28] also reported improved lung function and disease symptoms in individuals with asthma and COPD after switching from smoking to EC use. Three meta-analyses found an association between current, ever, and former EC use and the prevalence of asthma [23,25,31] and COPD [23]. The authors underlined the limited interpretation of their findings due to the cross-sectional nature of studies, observed heterogeneity, and likely publication bias.

##### Effects of EC Use by Smoking Status

- Tobacco-naïve EC users

One review [18] reported impaired lung function, measured by spirometry, in tobacco-naïve EC users compared to never users.

- EC-naïve smokers

One review reported that short-term EC use in smokers was associated with an increase in impedance and overall peripheral airway resistance, and a concomitant decrease in specific airway conductance, similar to TC use [21].

- Former TC smokers who switched to ECs

Some positive health changes (e.g., reduced carboxyhemoglobin levels) [19] and improvements in respiratory symptoms in former smokers who switched to regular EC use [21] were reported, although the authors recognized that the latter findings were flawed by selection bias and conflict of interest. One review [24] showed 40% lower odds of respiratory outcomes (including asthma, COPD, chronic bronchitis, and emphysema) for former smokers who switched to EC use compared to current exclusive smokers, based on a limited number of observational studies and unknown time of switching to ECs among former smokers.

- Dual (EC and TC) users

A positive association between dual use and the prevalence of asthma was reported in one systematic review [31], while a meta-analysis [25] found that the association with asthma prevalence was even stronger for dual use than for TC use alone. Temporality and a dose–response relationship were not determined due to the cross-sectional nature of the associations and lack of information on the intensity and duration of use. Another systematic review [23] concluded that EC use contributes independently to respiratory risk from TC use.

#### 3.3.2. In-Vivo Animal Studies

The NASEM report [16] found that exposure to EC aerosol and intratracheal exposure to e-liquids was associated with increased airway hyper reactivity, abnormalities in pulmonary function, significant increase in interleukin-1β (IL-1β), changes in immunomodulatory cytokines in the airways, and increases in markers of inflammation in mice. The authors noted substantial methodological differences, such as variations in EC devices, pumps, solutions, and exposures, which limit the comparability of results. Moreover, the reviewed studies failed to consider confounding factors, such as aerosol temperature and particle size.

One review [27] found that acute exposure to nicotine-containing EC aerosols induced higher inflammatory cell influx and release of pro-inflammatory cytokines, while a sub-acute exposure showed impairment of inflammatory response in mice. Chronic inhalation resulted in the downregulation of innate immunity against viral pathogens. In another review [18], long-term EC aerosol exposure was reported to have led to reduced small airway function, emphysematous lung destruction, and transient bronchoconstriction in animal models.

#### 3.3.3. In Vitro Cell Studies

Reviews suggest that EC aerosol exposure may cause changes in bronchial gene expression in human bronchial epithelial cells, similar to the effects from tobacco smoke [20,21].

### 3.4. Carcinogenic Effects

Five reviews considered carcinogenic effects of EC use [16,18,19,20,21]. The duration of EC exposure for carcinogenic outcomes was not clearly defined. The NASEM report [16] found no evidence that EC use is associated with intermediate cancer endpoints in humans, both when compared with TC use and no use of tobacco products. Further, the report found limited evidence that the EC aerosol can have mutagenic effects or damage DNA in human and animal in vitro cell models. Other reviews indicated some potentially carcinogenic effects based on compounds found in human urine samples of EC users [18,21] and likely increased the risk of lung cancer in high-risk individuals based on in vitro evidence [19,20].

## 4. Discussion

Our umbrella review synthesized the evidence from systematic reviews examining the cardiovascular, respiratory/pulmonary, and carcinogenic effects of EC use from human, animal, and in vitro studies. Reviewed evidence from human studies suggests the potential for cardiovascular harm of EC use through acute increases in heart rate, systolic and diastolic blood pressure, endothelial dysfunction, arterial stiffness, and biomarkers of oxidative stress. The effects were seen both in TC smokers and non-smokers. Evidence of the association of long-term EC use and chronic changes in heart rate, blood pressure, cardiac geometry and function, and increased risk of cardiovascular events compared to non-use was insufficient.

For pulmonary health outcomes, short-term use of ECs was found to reduce lung defense mechanisms and impact lung function and overall peripheral airway resistance both in smokers and non-smokers, while long-term EC use was suggested to increase exacerbations in individuals with asthma. Switching from chronic TC smoking to long-term EC use showed a potential reduction in cardiovascular and pulmonary/respiratory harm, especially in individuals with asthma and COPD. Evidence on the effects of dual EC and TC use compared to using one product alone is limited but suggests that EC use could be an independent risk factor contributing to respiratory harm. For carcinogenic outcomes, there is limited evidence of mutagenic effects or DNA damage in humans, and no evidence of an association between EC use and intermediate or long-term cancer endpoints.

Nicotine constituents of ECs were often found to be responsible for the cardiovascular [15,16,18,22,29,30], respiratory [16,19,29], and potentially carcinogenic effects [21]. Our findings are consistent with a previous umbrella review [6], which concluded that EC use is associated with increased cardiovascular risk, although to a lesser extent than combustible cigarettes. In addition, our umbrella review found that while EC use is associated with respiratory harm, there is evidence that switching from chronic TC to EC use likely reduces the harmful impact on lung function and improves disease symptoms in individuals with asthma and COPD.

Our study highlights important methodological weaknesses of the existing systematic reviews. First, the assessment of methodological quality and risk of bias using the AMSTAR-2 checklist [11] revealed several limitations in the methodology or reporting of existing systematic reviews, particularly in the items considered by AMSTAR-2 as critically important. Only about 20% of the included systematic reviews reported having developed a written protocol prior to conducting the review, despite recommendations by Cochrane Collaboration [32], the National Academy of Medicine (formerly known as the Institute of Medicine) [33], and PRISMA guidelines [34] since 2009.

An appropriate technique for systematic assessment of the quality and risk of bias, as recommended by the PRISMA Guidelines [10], was applied in only about 50% of reviews. Finally, only 10% of reviews provided a list of excluded studies. Notably, this recommendation was only added to the PRISMA Checklist in 2020. These findings are largely in line with existing evidence that despite several guidelines for conducting and reporting systematic reviews, less than a third of systematic reviews report having adhered to these guidelines [35]. The overall poor reporting across systematic reviews presents an important limitation in the current research on EC health effects. Systematic reviews should follow a structured and pre-defined process with rigorous methods to produce reliable findings that can help guide regulatory decisions.

Significant variations were also discovered in the timeframe of the examined health outcomes across the reviews. While some categorized health outcomes into acute and chronic, depending on the time-point of the health outcome assessment after EC exposure, others focused on the duration of EC use. For acute effects, if specified, the range spanned from minutes to hours, while for chronic effects, a much wider time range spanning from several days to several years was used, raising concerns regarding whether health effects assessed in a short span of several days can even be regarded as chronic.

Often, results were categorized into short-term and long-term outcomes without clear definitions, thereby, limiting the comparability of results across reviews. Moreover, considering that the ECs are relatively new to the tobacco marketplace, their true long-term health effects may not be observable until the middle of this century [36]. Perhaps the most important limitation of research in human health effects is that few systematic reviews distinguished health effects of EC use in smokers or former smokers, while many did not report the smoking status of study participants.

Since many EC users continue to smoke TCs [7] and may be at increased risk of cardiovascular disease compared to only cigarette smokers [37,38], the smoking status of study participants is needed to distinguish the impacts of EC from that of TC or dual use. Moreover, accounting not only for smoking status but also for smoking exposure with measures, such as intensity, duration, or pack-years, is needed in studies of EC health effects to properly estimate their independent risk [39]. Further, reviews generally did not account for the health status of human subjects. This presents an important issue, especially when estimating the health effects in those who transitioned from regular TC to EC use, as switching from combustible to EC use is often driven by poor health and existing health conditions.

A rapidly changing landscape of available EC devices and e-liquids imposes another major limitation of current research [40], particularly regarding the assessment of longer-term health effects. Underreporting EC device and e-liquid types was a critical limitation across most reviews, limiting the ability to compare health effects across different generations of EC devices and their e-liquid characteristics. Some reviews collected the data but did not utilize it for data analysis and interpretation. No reviews reported the effects of the more popular latest generation EC devices, such as JUUL, which appear to deliver nicotine in similar concentrations as combustible tobacco products [41].

In addition, each study only considered a specific set of EC brands, device types, and e-liquids and one mode of operation of the particular device, thereby, limiting the generalizability of their findings to other brands, e-liquids, and devices. Future systematic reviews should focus on clinical trials to minimize the variability in product devices, e-liquids, individual product use patterns, and study designs across the included studies. We expect that clinical trials will provide more potential for developing well-defined outcomes.

In addition to the shortcomings of the systematic reviews identified in this umbrella review, some common limitations of the primary studies were reported in the included reviews. Human experimental studies that compared the health effects of ECs to those of traditional smoking often used inconsistent measurements of pre/post plasma nicotine levels in the two groups. In addition, those less experienced with vaping may have inhaled a lower volume of aerosol and thus experienced a lesser acute health impact.

In studies that assessed the health impact in smokers who switched to ECs, the study population often included those aiming to quit smoking along with those with no intention to quit, which could have influenced compliance in abstaining from smoking during the studies. Long-term smokers switching to EC use were often reported to have continued smoking during the study period, which may have confounded the outcomes of these studies. Further, potential and apparent conflicts of interest (COI) were reported in a substantial number of original studies due to funding by EC manufacturers. Such studies tended to draw conclusions supportive of EC use compared to studies without COI [15,21].

Our umbrella review is subject to limitations. First, our analysis included only those literature reviews that explicitly self-identified as systematic in the title, abstract, keyword, or methods. This method was selected as the most straightforward way to detect a systematic review. Nevertheless, it led to the exclusion of some widely-cited reviews [42,43,44] that did not claim to be systematic, while our umbrella review suggests that many of the included reviews should not be considered systematic due to their methodological shortcomings indicated by the AMSTAR-2 checklist.

However, although AMSTAR-2 was considered most suitable among existing quality appraisal tools for our review [12], it has been previously found to set very high standards for its quality classifications [14,45,46,47]. A different quality appraisal tool may have yielded different results. The insufficient reporting of the effect sizes across the included systematic reviews precluded us from summarizing the evidence in quantitative terms. Further, our umbrella review included systematic reviews without imposing a restriction on publication date.

Although most reviews were published in the past four years, three were published prior to 2017, when evidence of health effects of ECs was more limited and dependent on earlier-generation devices. In addition, our review included evidence from in vitro and animal studies, which should be extrapolated with caution since laboratory exposure to EC aerosol may not accurately reflect real-life exposure in humans. Finally, original studies included in examined systematic reviews may have overlapped, thereby, overrepresenting findings of such studies in our umbrella review.

## 5. Conclusions

This umbrella review highlights the need for future systematic reviews with better adherence to established reporting guidelines. Such guidelines include developing and registering a peer-reviewed review protocol before the commencement of the review, adequately assessing the methodological quality and risk of bias in individual studies, and carefully examining the impact that potential conflicts of interest in individual studies may have on the outcomes. In addition, future reviews should adhere to a consistent definition of the duration of EC exposure (i.e., explicitly defined acute and long-term use) and of the device and e-liquid type, and should focus on the health effects of newer generation EC devices.

In reviews of human studies, EC use and the TC smoking status of participants should be systematically reported to distinguish the risks of vaping from those of smoking. When possible, adjustment for health status and cumulative history of smoking should be conducted. Meeting each of these needs will ensure that the evidence of the health consequences of EC use is clear and reliable and can thereby be more directly useful in developing effective and proportionate tobacco regulatory and policy interventions designed to protect public health.

## Data Availability

Not applicable.

## Supplementary Materials

The following supporting information can be downloaded at: https://www.mdpi.com/article/10.3390/ijerph19159054/s1, File S1: The search strategy for the PubMed database; Table S1: List of excluded non-systematic literature reviews with relevant health outcomes; Table S2: Systematic reviews, meta-analyses, and included health outcome domains; Table S3: Included systematic reviews- extended table with results, conclusions and limitations.

## References

[1] U.S. Department of Health and Human Services (2010). How Tobacco Smoke Causes Disease: The Biology and Behavioral Basis for Smoking-Attributable Disease: A Report of the Surgeon General.

[2] Farsalinos K., Cibella F., Caponnetto P., Campagna D., Morjaria J.B., Battaglia E., Caruso M., Russo C., Polosa R. (2016). Effect of continuous smoking reduction and abstinence on blood pressure and heart rate in smokers switching to electronic cigarettes. Intern. Emerg. Med..

[3] Vansickel A.R., Cobb C.O., Weaver M.F., Eissenberg T.E. (2010). A clinical laboratory model for evaluating the acute effects of electronic “cigarettes”: Nicotine delivery profile and cardiovascular and subjective effects. Cancer Epidemiol. Biomark. Prev..

[4] Vansickel A.R., Eissenberg T. (2012). Electronic cigarettes: Effective nicotine delivery after acute administration. Nicotine Tob. Res..

[5] Yan X.S., D’Ruiz C. (2015). Effects of using electronic cigarettes on nicotine delivery and cardiovascular function in comparison with regular cigarettes. Regul. Toxicol. Pharmacol..

[6] Peruzzi M., Biondi-Zoccai G., Carnevale R., Cavarretta E., Frati G., Versaci F. (2020). Vaping Cardiovascular Health Risks: An Updated Umbrella Review. Curr. Emerg. Hosp. Med. Rep..

[7] Mayer M., Reyes-Guzman C., Grana R., Choi K., Freedman N.D. (2020). Demographic Characteristics, Cigarette Smoking, and e-Cigarette Use Among US Adults. JAMA Netw. Open.

[8] U.S. Department of Health and Human Services (2020). Smoking Cessation: A Report of the Surgeon General.

[9] Aromataris E., Fernandez R., Godfrey C.M., Holly C., Khalil H., Tungpunkom P. (2015). Summarizing systematic reviews: Methodological development, conduct and reporting of an umbrella review approach. Int. J. Evid.-Based Healthc..

[10] Page M.J., McKenzie J.E., Bossuyt P.M., Boutron I., Hoffmann T.C., Mulrow C.D., Shamseer L., Tetzlaff J.M., Akl E.A., Brennan S.E. (2021). The PRISMA 2020 statement: An updated guideline for reporting systematic reviews. BMJ.

[11] Shea B.J., Reeves B.C., Wells G., Thuku M., Hamel C., Moran J., Moher D., Tugwell P., Welch V., Kristjansson E. (2017). AMSTAR 2: A critical appraisal tool for systematic reviews that include randomised or non-randomised studies of healthcare interventions, or both. BMJ.

[12] Ma L.-L., Wang X., Yang Z.-H., Huang D., Weng H., Zeng X.-T. (2020). Methodological quality (risk of bias) assessment tools for primary and secondary medical studies: What are they and which is better?. Mil. Med. Res..

[13] Perry R., Whitmarsh A., Leach V., Davies P. (2021). A comparison of two assessment tools used in overviews of systematic reviews: ROBIS versus AMSTAR-2. Syst. Rev..

[14] De Santis K.K., Lorenz R.C., Lakeberg M., Matthias K. (2021). The application of AMSTAR2 in 32 overviews of systematic reviews of interventions for mental and behavioural disorders: A cross-sectional study. Res. Synth. Methods.

[15] Kennedy C., van Schalkwyk M.C., McKee M., Pisinger C. (2019). The cardiovascular effects of electronic cigarettes: A systematic review of experimental studies. Prev. Med..

[16] National Academies of Sciences, Engineering, and Medicine (2018). Public Health Consequences of E-Cigarettes.

[17] Gualano M.R., Passi S., Bert F., La Torre G., Scaioli G., Siliquini R. (2014). Electronic cigarettes: Assessing the efficacy and the adverse effects through a systematic review of published studies. J. Public Health.

[18] Bozier J., Chivers E.K., Chapman D.G., Larcombe A.N., Bastian N.A., Masso-Silva J.A., Byun M.K., McDonald C.F., Alexander L.E.C., Ween M.P. (2020). The Evolving Landscape of e-Cigarettes: A Systematic Review of Recent Evidence. Chest.

[19] Harrell P.T., Simmons V.N., Correa J.B., Padhya T.A., Brandon T.H. (2014). Electronic nicotine delivery systems (“e-cigarettes”): Review of safety and smoking cessation efficacy. Otolaryngol.-Head Neck Surg..

[20] Ioakeimidis N., Vlachopoulos C., Tousoulis D. (2016). Efficacy and Safety of Electronic Cigarettes for Smoking Cessation: A Critical Approach. Hell. J. Cardiol..

[21] Pisinger C., Døssing M. (2014). A systematic review of health effects of electronic cigarettes. Prev. Med..

[22] Skotsimara G., Antonopoulos A.S., Oikonomou E., Siasos G., Ioakeimidis N., Tsalamandris S., Charalambous G., Galiatsatos N., Vlachopoulos C., Tousoulis D. (2019). Cardiovascular effects of electronic cigarettes: A systematic review and meta-analysis. Eur. J. Prev. Cardiol..

[23] Wills T.A., Soneji S.S., Choi K., Jaspers I., Tam E.K. (2020). E-cigarette use and respiratory disorders: An integrative review of converging evidence from epidemiological and laboratory studies. Eur. Respir. J..

[24] Goniewicz M.L., Miller C.R., Sutanto E., Li D. (2020). How effective are electronic cigarettes for reducing respiratory and cardiovascular risk in smokers? A systematic review. Harm Reduct. J..

[25] Xian S., Chen Y. (2021). E-cigarette users are associated with asthma disease: A meta-analysis. Clin. Respir. J..

[26] Martinez-Morata I., Sanchez T.R., Shimbo D., Navas-Acien A. (2020). Electronic Cigarette Use and Blood Pressure Endpoints: A Systematic Review. Curr. Hypertens. Rep..

[27] Bravo-Gutiérrez O., Falfán-Valencia R., Ramírez-Venegas A., Sansores R., Ponciano-Rodríguez G., Pérez-Rubio G. (2021). Lung Damage Caused by Heated Tobacco Products and Electronic Nicotine Delivery Systems: A Systematic Review. Int. J. Environ. Res. Public Health.

[28] Glasser A.M., Collins L., Pearson J.L., Abudayyeh H., Niaura R.S., Abrams D.B., Villanti A.C. (2017). Overview of Electronic Nicotine Delivery Systems: A Systematic Review. Am. J. Prev. Med..

[29] Larue F., Tasbih T., Ribeiro P., Lavoie K.L., Dolan E., Bacon S.L. (2021). Immediate physiological effects of acute electronic cigarette use in humans: A systematic review and meta-analysis. Respir. Med..

[30] Garcia P.D., Gornbein J.A., Middlekauff H.R. (2020). Cardiovascular autonomic effects of electronic cigarette use: A systematic review. Clin. Auton. Res..

[31] Chand B.R., Hosseinzadeh H. (2021). Association between e-cigarette use and asthma: A systematic review and meta-analysis. J. Asthma.

[32] Henderson L., Craig J., Willis N., Tovey D., Webster A. (2010). How to write a Cochrane systematic review. Clin. Res. Nephrol..

[33] Institute of Medicine (2011). Finding What Works in Health Care: Standards for Systematic Reviews.

[34] Moher D., Liberati A., Tetzlaff J., Altman D.G., PRISMA Group (2009). Preferred reporting items for systematic reviews and meta-analyses: The PRISMA statement. BMJ.

[35] Page M., Shamseer L., Altman D.G., Tetzlaff J., Sampson M., Tricco A.C., Catalá-López F., Li L., Reid E.K., Sarkis-Onofre R. (2016). Epidemiology and Reporting Characteristics of Systematic Reviews of Biomedical Research: A Cross-Sectional Study. PLoS Med..

[36] Gotts J.E., Jordt S.E., McConnell R., Tarran R. (2019). What are the respiratory effects of e-cigarettes?. BMJ.

[37] Osei A.D., Mirbolouk M., Orimoloye O.A., Dzaye O., Uddin S.I., Benjamin E.J., Hall M.E., DeFilippis A.P., Stokes A., Bhatnagar A. (2019). Association Between E-Cigarette Use and Cardiovascular Disease Among Never and Current Combustible-Cigarette Smokers. Am. J. Med..

[38] Kim C.-Y., Paek Y.-J., Seo H.G., Cheong Y.S., Lee C.M., Park S.M., Lee K. (2020). Dual use of electronic and conventional cigarettes is associated with higher cardiovascular risk factors in Korean men. Sci. Rep..

[39] Sargent J.D., Halenar M.J., Edwards K.C., Woloshin S., Schwartz L., Emond J., Tanski S., A Taylor K., Pierce J.P., Liu J. (2022). Tobacco use and respiratory symptoms among adults: Findings from the Longitudinal Population Assessment of Tobacco and Health (PATH) Study 2014-16. Nicotine Tob. Res..

[40] Benowitz N.L., Fraiman J.B. (2017). Cardiovascular effects of electronic cigarettes. Nat. Rev. Cardiol..

[41] Goniewicz M.L., Boykan R., Messina C.R., Eliscu A., Tolentino J. (2018). High exposure to nicotine among adolescents who use Juul and other vape pod systems (‘pods’). Tob. Control.

[42] McNeil A., Brose L.S., Calder R., Hitchman S.C., Hajek P., McRobbie H. (2015). E-Cigarettes: An Evidence Update. A Report Commissioned by Public Health England.

[43] McNeill A., Brose L.S., Calder R., Bauld L., Robson D. (2018). Evidence Review of E-Cigarettes and Heated Tobacco Products 2018. A Report Commissioned by Public Health England.

[44] Royal College of Physicians (2016). Nicotine without Smoke: Tobacco Harm Reduction.

[45] Calati R., Filipponi C., Mansi W., Casu D., Peviani G., Gentile G., Tambuzzi S., Zoja R., Fornaro M., Lopez-Castroman J. (2021). Cancer diagnosis and suicide outcomes: Umbrella review and methodological considerations. J. Affect. Disord..

[46] Gold N., Yau A., Rigby B., Dyke C., Remfry E.A., Chadborn T. (2021). Effectiveness of Digital Interventions for Reducing Behavioral Risks of Cardiovascular Disease in Nonclinical Adult Populations: Systematic Review of Reviews. J. Med. Internet Res..

[47] Kim M.M., Pound L., Steffensen I., Curtin G.M. (2021). Reporting and methodological quality of systematic literature reviews evaluating the associations between e-cigarette use and cigarette smoking behaviors: A systematic quality review. Harm Reduct. J..

