# Supplementary material for: Health Effects of Electronic Cigarettes: An Umbrella Review and Methodological Considerations
Source: Int J Environ Res Public Health. 2022 Jul 25;19(15):9054. doi: 10.3390/ijerph19159054 (PMC9330875; doi:10.3390/ijerph19159054)
Supplement: Supplementary file 1 [file ijerph-19-09054-s001.zip › Supplementary File 1.pdf]

Health Effects of Electronic Cigarettes: An Umbrella Review and Methodological Considerations

Nargiz Travis, MSPH[1], Marie Knoll, MSPH[1], Christopher J. Cadham, MPH[2], Steven Cook, PhD[3], Kenneth E. Warner, PhD[2], Nancy L. Fleischer, PhD[3], Clifford E. Douglas, JD[2], Luz María Sánchez-Romero, PhD[1], Ritesh Mistry, PhD[4], Rafael Meza, PhD[3], Jana L. Hirschtick, PhD[3], David T. Levy, PhD[1].

[1]Lombardi Comprehensive Cancer Center, Georgetown Medical University, Washington, DC

[2]Department of Health Management and Policy, School of Public Health, University of Michigan, Ann Arbor, MI

[3]Department of Epidemiology, School of Public Health, University of Michigan, Ann Arbor, MI

[4]Department of Health Behavior and Health Education, School of Public Health, University of Michigan, Ann Arbor, MI

Supplementary File 1. The search strategy for the PubMed database.

Terminology: ("electronic cigarette" OR "e-cigarette" OR "electronic nicotine delivery system" OR "personal vaporiser" OR "personal vaporizer" OR (“e-liquid” AND “nicotine content”)) AND (“systematic review” OR “meta-analysis” OR “review”)

Date: Through January 25, 2022

Limit: None

Results: 717
