# Supplementary material for: Health Effects of Electronic Cigarettes: An Umbrella Review and Methodological Considerations
Source: Int J Environ Res Public Health. 2022 Jul 25;19(15):9054. doi: 10.3390/ijerph19159054 (PMC9330875; doi:10.3390/ijerph19159054)
Supplement: Supplementary file 1 [file ijerph-19-09054-s001.zip › Supplementary Table 1. Excuded Reviews.pdf]

Health Effects of Electronic Cigarettes: An Umbrella Review and Methodological Considerations

Nargiz Travis, MSPH[1], Marie Knoll, MSPH[1], Christopher J. Cadham, MPH[2], Steven Cook, PhD[3], Kenneth E. Warner, PhD[2], Nancy L. Fleischer, PhD[3], Clifford E. Douglas, JD[2], Luz María Sánchez-Romero, PhD[1], Ritesh Mistry, PhD[4], Rafael Meza, PhD[3], Jana L. Hirschtick, PhD[3], David T. Levy, PhD[1].

[1]Lombardi Comprehensive Cancer Center, Georgetown Medical University, Washington, DC

[2]Department of Health Management and Policy, School of Public Health, University of Michigan, Ann Arbor, MI

[3]Department of Epidemiology, School of Public Health, University of Michigan, Ann Arbor, MI

[4]Department of Health Behavior and Health Education, School of Public Health, University of Michigan, Ann Arbor, MI

Supplementary Table 1. List of excluded non-systematic literature reviews with relevant health outcomes.

First author, year	Title
Bals et al., 2019	Electronic cigarettes: a task force report from the European Respiratory Society. *DOI:10.1183/13993003.01151-2018*
Eltorai et al., 2019	Impact of Electronic Cigarettes on Various Organ Systems. *DOI: 10.4187/respcare.06300*
McNeill et al., 2018	Evidence review of e-cigarettes and heated tobacco products 2018. A report commissioned by Public Health England. *Accessible at https://www.gov.uk/government/publications/*
Jankovski et al., 2017	E-smoking: Emerging public health problem? *DOI: 10.13075/ijomeh.1896.01046*
Qasim et al., 2017	Impact of Electronic Cigarettes on the Cardiovascular System. *DOI: 10.1161/JAHA.117.006353*
Zhang et al., 2017	Safety Assessment of Electronic Cigarettes and Their Relationship with Cardiovascular Disease. *DOI: 10.3390/ijerph15010075*
Breland et al., 2017	Electronic cigarettes: what are they and what do they do? *DOI: 0.1111/nyas.12977*
Royal College of Physicians, 2016	Nicotine without smoke: Tobacco harm reduction. *Accessible at https://www.rcplondon.ac.uk/projects/outputs/nicotine-without-smoke-tobacco-harm-reduction*
McNeill et al., 2015	E-cigarettes: an evidence update. A report commissioned by Public Health England. *Accessible at https://www.gov.uk/government/publications/e-cigarettes-*

	an-evidence-update
Nelluri et al., 2015	The current literature regarding the cardiovascular effects of electronic cigarettes. *DOI: 10.2217/fca.15.8*
Born et al., 2015	Electronic cigarettes: A primer for clinicians. *DOI: 10.1177/019459981558575*
Callahan-Lyon 2014	Electronic cigarettes: human health effects. *DOI: 10.1136/tobaccocontrol-2013-051470*
Middlekauff et al., 2014	Adverse effects of cigarette and noncigarette smoke exposure on the autonomic nervous system. *DOI: 10.1016/j.jacc.2014.06.1201*
Hajek et al., 2014	Electronic cigarettes: review of use, content, safety, effects on smokers and potential for harm and benefit. *DOI: 10.1111/add.12659.*
