# Supplementary material for: Health Effects of Electronic Cigarettes: An Umbrella Review and Methodological Considerations
Source: Int J Environ Res Public Health. 2022 Jul 25;19(15):9054. doi: 10.3390/ijerph19159054 (PMC9330875; doi:10.3390/ijerph19159054)
Supplement: Supplementary file 1 [file ijerph-19-09054-s001.zip › Supplementary Table 2. Health outcome domains.pdf]

Health Effects of Electronic Cigarettes: An Umbrella Review and Methodological Considerations

Nargiz Travis, MSPH[1], Marie Knoll, MSPH[1], Christopher J. Cadham, MPH[2], Steven Cook, PhD[3], Kenneth E. Warner, PhD[2], Nancy L. Fleischer, PhD[3], Clifford E. Douglas, JD[2], Luz María Sánchez-Romero, PhD[1], Ritesh Mistry, PhD[4], Rafael Meza, PhD[3], Jana L. Hirschtick, PhD[3], David T. Levy, PhD[1].

[1]Lombardi Comprehensive Cancer Center, Georgetown Medical University, Washington, DC

[2]Department of Health Management and Policy, School of Public Health, University of Michigan, Ann Arbor, MI

[3]Department of Epidemiology, School of Public Health, University of Michigan, Ann Arbor, MI

[4]Department of Health Behavior and Health Education, School of Public Health, University of Michigan, Ann Arbor, MI

Supplementary Table 2. Systematic reviews, meta-analyses and included health outcome domains.

	Cardiovascular	Respiatory/Pulmonary	Carcinogenic
Larue et al., 2021§	X	X	
Bravo-Gutierrez et al., 2021		X	
Chand & Hosseinzadeh, 2021§		X	
Xian & Chen, 2021§		X	
Wills et al., 2021§		X	
Garcia et al., 2020	X		
Bozier et al., 2020	X	X	X
Martinez-Morata et al., 2020	X		
Goniewicz et al., 2020	X	X	
Skotsimara et al., 2019§	X		
Kennedy et al., 2019	X		
NASEM, 2018	X	X	X
Glasser et al., 2017	X	X	
Ioakeimidis et al., 2016	X	X	X
Pisinger & Dossing, 2014	X	X	X
Harrell et al., 2014	X	X	X
Gualano et al., 2014		X	

§ Meta-analysis.
